# Tetrabromobisphenol A Is an Efficient Stabilizer of the Transthyretin Tetramer

**DOI:** 10.1371/journal.pone.0153529

**Published:** 2016-04-19

**Authors:** Irina Iakovleva, Afshan Begum, Kristoffer Brännström, Alexandra Wijsekera, Lina Nilsson, Jin Zhang, Patrik L. Andersson, A. Elisabeth Sauer-Eriksson, Anders Olofsson

**Affiliations:** 1 Department of Medical Biochemistry and Biophysics, Umeå University, Umeå, Sweden; 2 Department of Chemistry, Umeå University, Umeå, Sweden; University of Florida, UNITED STATES

## Abstract

Amyloid formation of the human plasma protein transthyretin (TTR) is associated with several human disorders, including familial amyloidotic polyneuropathy (FAP) and senile systemic amyloidosis. Dissociation of TTR’s native tetrameric assembly is the rate-limiting step in the conversion into amyloid, and this feature presents an avenue for intervention because binding of an appropriate ligand to the thyroxin hormone binding sites of TTR stabilizes the native tetrameric assembly and impairs conversion into amyloid. The desired features for an effective TTR stabilizer include high affinity for TTR, high selectivity in the presence of other proteins, no adverse side effects at the effective concentrations, and a long half-life in the body. In this study we show that the commonly used flame retardant tetrabromobisphenol A (TBBPA) efficiently stabilizes the tetrameric structure of TTR. The X-ray crystal structure shows TBBPA binding in the thyroxine binding pocket with bromines occupying two of the three halogen binding sites. Interestingly, TBBPA binds TTR with an extremely high selectivity in human plasma, and the effect is equal to the recently approved drug tafamidis and better than diflunisal, both of which have shown therapeutic effects against FAP. TBBPA consequently present an interesting scaffold for drug design. Its absorption, metabolism, and potential side-effects are discussed.

## Introduction

Transthyretin (TTR) is a homotetrameric plasma protein involved in the transport of thyroxine hormone (T4) and retinol-binding protein. The protein is mainly produced in the liver, but expression is also found in the choroid plexus of the brain and within the retina of the eye [1]. TTR has amyloidogenic features and is associated with several different disorders, including familial amyloidotic polyneuropathy (FAP) [2–4], familial amyloidotic cardiomyopathy [5], and senile systemic amyloidosis [6,7]. TTR amyloid has also recently been suggested to play a role in spinal lumbar stenosis [8] and preeclampsia [9,10]. The formation of amyloid from TTR is initiated through dissociation of its native tetramer, which is the rate-limiting step in the conversion of the native protein into amyloid [11,12].

Familial variants of TTR amyloidosis are frequently treated by liver transplantation such that the mutated protein is exchanged for the wild-type version. This treatment slows down the progression of the disease and should therefore be initiated as early as possible after the onset of symptoms [13–15]. However, it must be noted that liver transplantation is a major surgical undertaking and is associated with risks of complications due to compromised heart function [13].

Several steps are involved in the conversion from a native TTR protein to a misfolded protein. Dissociation of the tetramer is the rate-limiting step, and this step also provides an avenue for intervention. TTR has two binding sites for the T4 hormone situated at the dimer-dimer interface of the tetramer, and binding of the hormone has been found to reduce the tetrameric dissociation rate and consequently maintain TTR in its tetrameric state and prevent amyloid formation [16].

The saturation level of T4 to TTR within the blood is only a few percent, which is too low to effectively stabilize the tetramer, and external administration of the hormone to reach full saturation of all TTR molecules *in vivo* cannot be done without adverse side effects [17]. Thus alternative stabilizing agents have been developed [18–33], and the drug tafamidis (Vyndaqel^™^) has been approved for clinical use [4].

An effective TTR stabilizer should have high affinity for the hormone binding site and should display high selectivity for its target in the human body. It should moreover have a low incidence of side effects and should be resistant to enzymatic degradation *in vivo*. Nonspecific binding to other components greatly reduces the efficacy of many drugs. We have recently shown that the selectivity of TTR binders in human plasma varies considerably and that many drugs, despite a very high affinity for TTR *in vitro*, frequently require a very high stoichiometric excess relative to the TTR protein in order to effectively maintain the tetrameric form of TTR [34]. To have a stabilizing effect on TTR, the concentration of the ligand in the plasma must be above the plasma concentration of tetrameric TTR, which is normally around 4 μM [35]. Moreover, the stability of the drug *in vivo* has to be long enough to ensure a reasonable number of dosages per day, and the stability should be such that a steady-state level of the drug can be reached over time.

A number of organohalogen compounds, including brominated flame retardants, have been reported to bind to human TTR *in vitro* [36,37]. One of these– 2,2’,6,6’-tetrabromo-4,4’-isopropylidentediphenol (Tetrabromobisphenol A, TBBPA)–is produced in large quantities for use as a reactive flame retardant in the laminate coating of printed circuit boards and as an additive flame retardant in acrylonitrile butadiene styrene (ABS) plastics, and it has a world market of around 170,000 metric tons annually [36,38–40].

In the present work, we have investigated the binding of TBBPA to TTR, and we show that TBBPA effectively prevents the dissociation of TTR tetramers *in vitro* in both low and neutral pH environments. Using a recently developed assay to evaluate the selectivity of TTR-stabilizing drugs in human plasma [34], we show that TBBPA exhibits an extraordinarily high selectivity and that an essentially complete stabilization is observed already at a stoichiometric ratio. This is significantly better than diflunisal and is within the same range as the recently approved and highly selective drug tafamidis, both of which have been shown to have beneficial clinical effects in humans [41–43].

TBBPA consequently presents an interesting scaffold in the quest to design improved TTR stabilizers, and its properties with respect to administration, metabolism, and potential side-effects are discussed.

## Results

### TBBPA significantly decreases the TTR dissociation rate under acidic denaturation conditions

**Fig 1:**
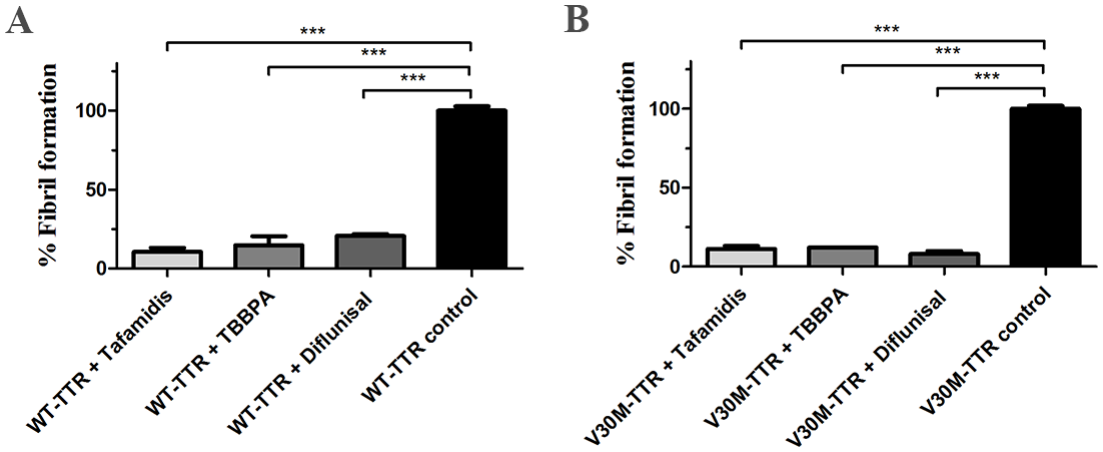
TBBPA prevents tetramer dissociation under acidic conditions. WT-TTR (A) and V30M-TTR (B) dissolved in PBS at a tetrameric concentration of 15 μM were pre-incubated alone or in the presence of TBBPA, diflunisal, or tafamidis (each at 15 μM) for 2 h. Aggregation of WT-TTR and V30M-TTR was initiated by lowering the pH to 4.5 using a sodium-acetate/acetic acid buffer followed by incubation for 72 h. The percentage of aggregation relative to the control was monitored by measuring the turbidity at 400 nm. Statistical analysis was performed using one-way ANOVA, and data are presented as the mean ± standard deviation of the percent fibril formation (*n* = 2, *** *p* < 0.001).

Amyloid formation by TTR can be induced at low pH, which results in disruption of the tetramer and the subsequent formation of aggregates. This event can be monitored by a simple turbidity measurement. Diflunisal and tafamidis have both been previously studied for their ability to stabilize TTR tetramers [11,44], and they are included in this study only for comparison purposes. Turbidity measurements showed that TBBPA efficiently prevents the aggregation of TTR as well as the clinically relevant TTRVal30Met variant at low pH in a similar manner to diflunisal and tafamidis (Fig 1).

### Isothermal titration calorimetry (ITC)

The binding affinity of TBBPA to TTR was determined using ITC according to standard procedures. Assuming independent binding sites, TBBPA binds to TTR with a K_d_ of 20 nM. The binding affinities are displayed in Table 1, which also includes the K_d_ values of diflunisal and tafamidis adapted from [34] and [11], respectively.

**Table 1:** K_d_ values obtained by ITC experiments and the IC_50_ values in plasma for TBBPA, tafamidis, and diflunisal.

*Substance name*	*Binding affinity to TTR (K*_*d*_*) nM*	*IC*_*50*_ *plasma (μM)*
TBBPA	20	3.3 ± 0.5
Tafamidis	^a^3	4.8 ± 0.6
Diflunisal	^b^580	^b^25.0 ± 12.0

^a^ IC_50_ value in plasma for tafamidis [11].

^b^ IC_50_ value in plasma diflunisal [34].

### TBBPA prevents TTR toxicity in neuroblastoma cells

**Fig 2:**
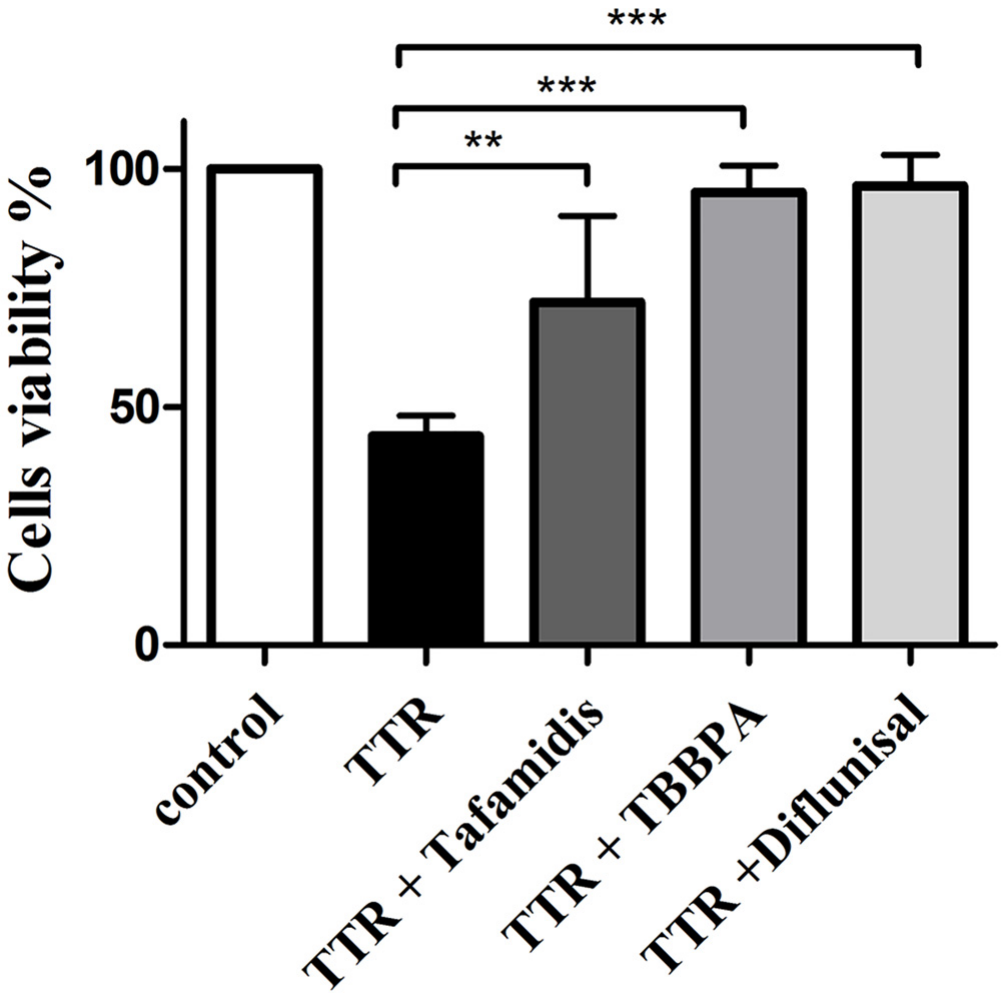
Probing the ability of the compounds to prevent TTR-mediated cytotoxicity in a human neuronal cell line. TTR at a final tetrameric concentration of 15 μM was pre-incubated with TBBPA, diflunisal, or tafamidis (15 μM each) for 2 h. TTR or the TTR with inhibitor complexes were added to SH-SY5Y cells and incubated for 72 h. Cell viability was measured with a resazurin assay [46]. Data are reported as the mean cell viability ± the standard deviation (*n* = 3, ** *p* < 0.01; *** *p* < 0.001). The addition of TTR tetramer stabilizers leads to a statistically significant increase in the number of viable cells compared to cells exposed to TTR alone. The statistical significance was assessed using one-way ANOVA.

It is known that preservation of tetrameric integrity also impairs the ability of TTR to exert a cytotoxic effect [45]. To evaluate the ability of TBBPA to suppress TTR-induced toxicity, we used a cell-viability assay based on human SH-SY5Y neuroblastoma cells [46]. TTR tetramers at 15 μM were incubated with cells and with or without compounds for 72 h. The cytotoxic effect of TTR was monitored by a decrease in the conversion of resazurin to resorufin. Incubation with TTR alone resulted in a 65% reduction in cell survival, and the addition of equimolar concentrations (15 μM) of TBBPA, tafamidis, or diflunisal increased cell survival to essentially that of the untreated control cells (Fig 2).

### TBBPA is selective in human plasma

**Fig 3:**
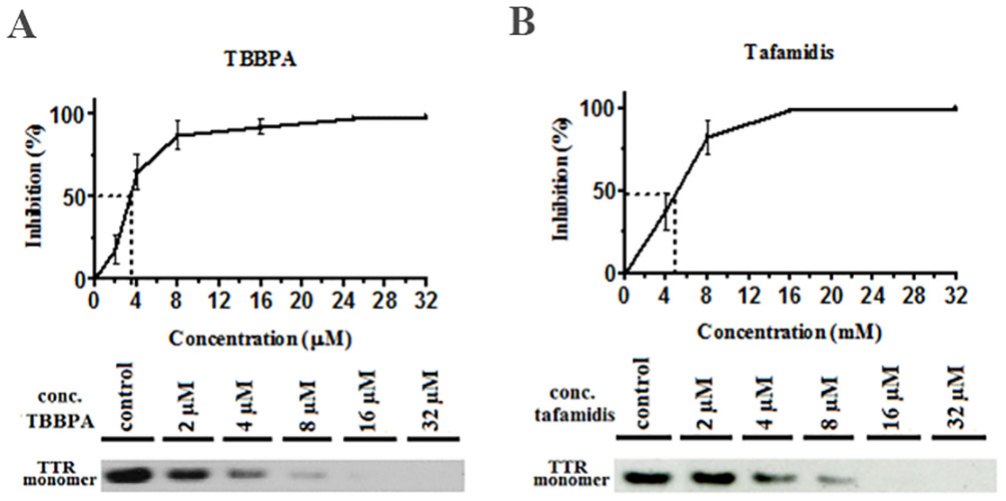
Determining the selectivity of TBBPA and tafamidis for TTR in human plasma. TBBPA and tafamidis were titrated at different concentrations in human plasma from a single healthy donor and incubated for 2 h. The dissociation of plasma TTR was initiated by the addition of urea at a final concentration of 4.0 M. The dissociation to monomeric TTR was monitored by western blot and quantified with the ImageJ 2.0 software. The inhibitory concentration at 50% in plasma (IC_50_) was estimated from three independent experiments. (A) TBBPA. (B) Tafamidis.

The ability of a drug to stabilize the tetrameric form of TTR by binding to the hydrophobic hormone binding pockets at the dimer interface of the tetramer is directly correlated to the drug’s binding affinity. However, the effective concentration of the drug can be compromised by nonspecific binding to other components that are present in plasma, and this can result in poor selectivity. We have recently developed an assay that determines the ability of a drug to stabilize TTR in the presence of human plasma. The tetrameric concentration of TTR *in vivo* is approximately 5 μM (0.28 mg/ml), and this sets the lower concentration limit for the drug. Using this assay, we found that TBBPA is highly specific and effectively stabilizes TTR at a stoichiometric ratio in plasma (Fig 3A). The highly selective drug tafamidis was included for comparison and is shown in Fig 3B.

The inhibitory concentration at 50% in plasma (IC_50_) shows that the approved drug tafamidis and TBBPA have high selectivity with IC_50_ values of 4.8 ± 0.6 μM and 3.3 ± 0.5 μM, respectively (Table 1). The IC_50_ for diflunisal (25.0 μM) was obtained from the recently published work by Iakovleva and et al. [34].

### Crystal structure of the TTR—TBBPA complex

**Fig 4:**
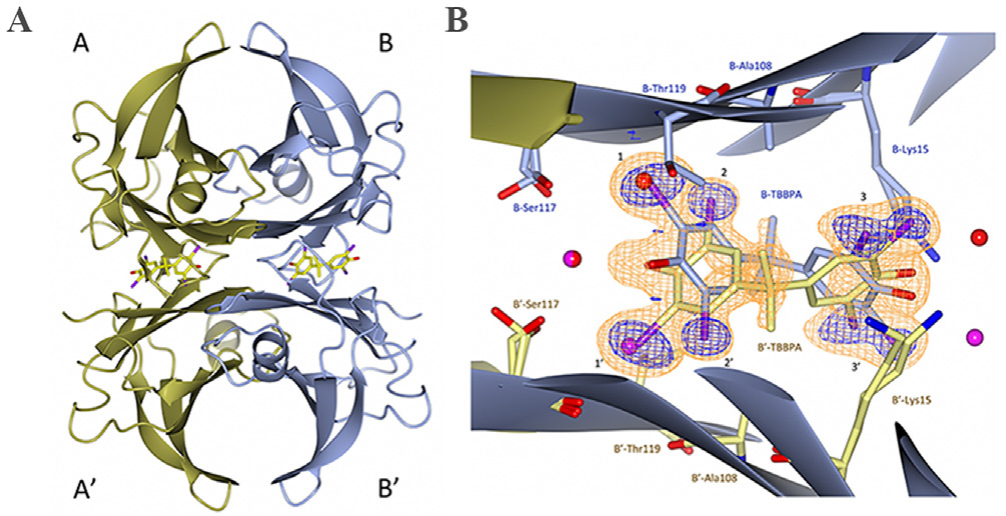
(A) The TTR-TBBPA structure shows the orientation of the ligand within the T4 binding sites. (B) Close-up view of the dimer-dimer interface of monomers B and B'. The σA-weighted (m|Fo|−D|Fc|) electron density is contoured at 3 times the root-mean-square value of the map and is shown in orange. To reduce model bias, the TBBPA molecules were excluded from the coordinate file that was subjected to one round of simulated annealing before calculation of the electron density map. The anomalous log-likelihood-gradient (LLG) map shown in dark blue shows the positions of the eight symmetry-related bromine atoms and verifies the modeled orientation of the TBBPA compound in the binding site. HBP1–3 and HBP1'–3' are indicated with numbers.

The T4 molecule contains four iodines. Based on their orientation within the hormone binding site of TTR in the previously solved co-crystal structure, the hormone binding site was divided into an inner and outer cavity comprising three symmetry-related pairs of halogen binding pockets, HBP1 (HBP1'), HBP2 (HBP2'), and HBP3 (HBP3') [29]. The side chains of Met13, Lys15, and Thr106 define the outer pocket HBP1. The middle pocket, HBP2, is composed of the hydrophobic side chains of residues Lys15, Leu17, Ala109, and Leu110, while the main-chain carbonyl groups of Lys15, Ala108, and Ala109 form a hydrophilic surface. The innermost pocket, HBP3, is formed by residues Ala108, Ala109, Leu110, Ser117, Thr118, and Thr119. Like HBP2, HBP3 has a hydrophilic surface consisting of the main-chain carbonyl oxygens and the amino groups of Ala108, Ala109, Leu110, and Thr118 and the polar parts of the Ser117 and Thr119 side chains. In this work, we have co-crystallized human wild-type TTR with TBBPA and solved the structure at a resolution 1.4 Å. Our structure shows that two symmetric TBBPA molecules are buried within the tetramer interface where symmetry-related bromine atoms occupy the three halogen binding pockets, HBP1, HBP2, and HBP3, in the T4 hormone binding site (Fig 4).

**Fig 5:**
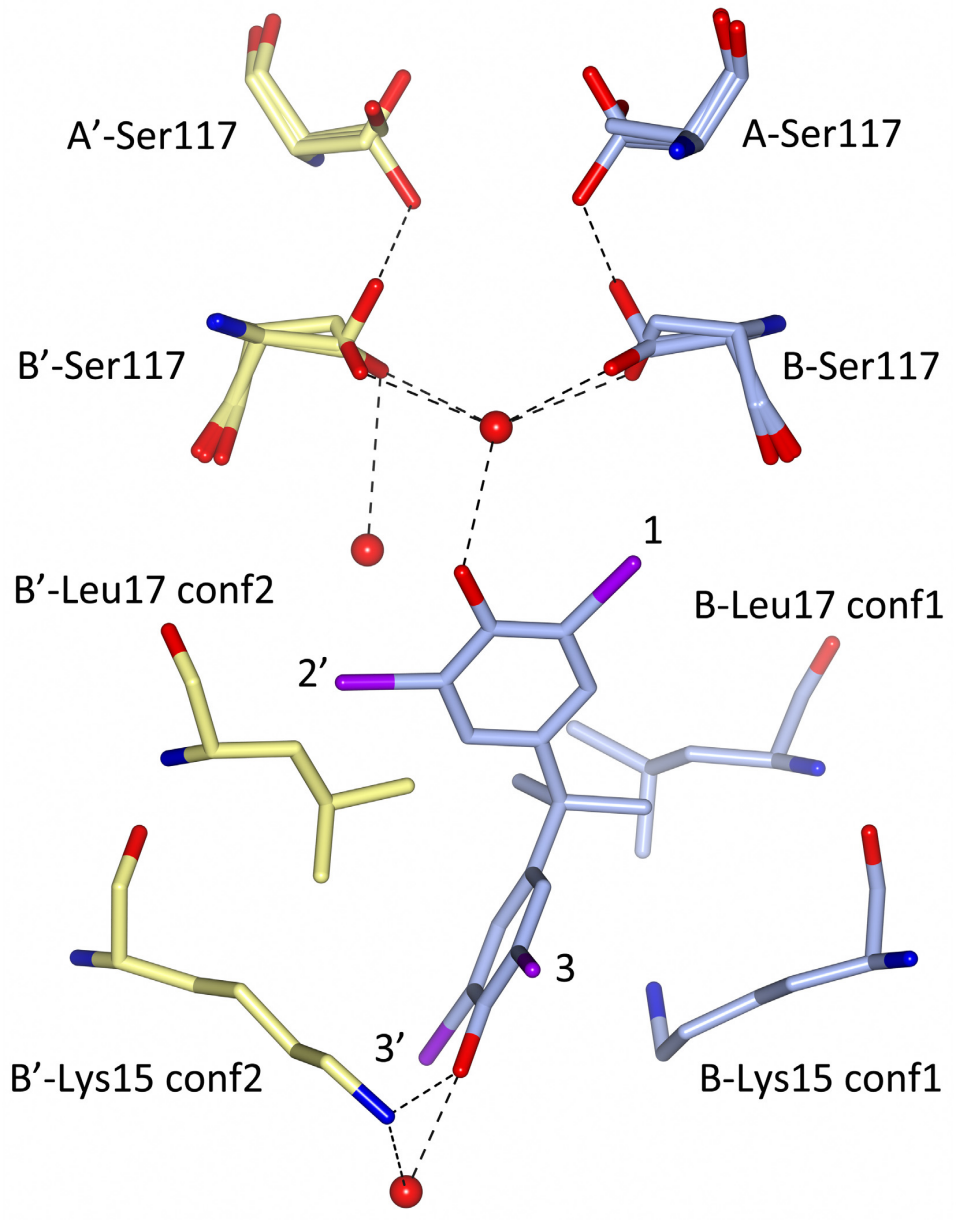
Molecular interaction between TBBPA and TTR at the B—B' hormone-binding site. Carbon atoms from the B monomer and TBBPA are colored in blue, and carbon atoms from the B' monomer are colored in yellow. Lys15 and Leu17 have two rotamer conformations (conf1 and conf2), of which only the ones interacting with the bound TBBPA ligand are shown. The Nz atom of the B'-Lys15 side chain forms a hydrogen bond with a water molecule and the O5 atom of the TBBPA ligand, whereas the Nz atom of B-Lys15 adopts a different orientation to allow for a hydrophobic interaction with the TBBPA benzene ring. The side chain of Ser117 has three rotamer conformations of which one (occupancy 25%) makes a hydrogen bond to the same residue in the A monomer. The HBP1–3 and HBP1'–3' binding sites are indicated with black numbers, and bromine atoms are colored in purple.

The bend of the TBBPA molecule at the central gem-dimethyl group is accommodated in the T4 binding site by a ligand-induced rotamer conformational change of the Leu17 side chain (Fig 5). Furthermore, the position of the hydroxyl group on the outer side of the cavity (HBP3 and HBP3') is stabilized by a hydrogen bond to Lys15 that also occupies two rotamer conformations in the structure (Fig 5).

## Discussion

From *in vitro* and cell-based experiments, it is well established that the rate of TTR tetramer dissociation determines TTR’s propensity to aggregate and that tetramer dissociation also correlates with its cytotoxic propensity [11,43,47]. The exact species of TTR causing the tissue damage is still not fully known, and although massive amyloid depositions can be detrimental to specific organs, biopsies of peripheral nerves from FAP patients as well as cell studies suggest that oligomeric TTR assemblies and possibly even monomers might be a cause of the tissue damage [47–52]. The strong correlation between dissociation of the tetramer, the development of a cytotoxic response, and formation of amyloid suggests that stabilization of the TTR tetramer is an attractive therapeutic approach irrespective of the downstream events.

One of the primary requirements for a drug to stabilize TTR *in vivo* is that it must be constantly present at a concentration in plasma that saturates the majority of the TTR molecules. TTR has two ligand binding sites, but depending on the ligand these frequently display a strong negative cooperativity, for example, the binding affinities between the first and the second T4 ligands differ by a factor of around 100 [53]. This implies that the second ligand will only contribute by 1% of the total stabilizing energy and consequently that most of the stabilizing effect is due to the binding of a single ligand. Given that TTR generally has a concentration *in vivo* around 4 μM, this implies that the drug has to be present at a concentration of at least 4 μM to effectively stabilize TTR. This also means that to acquire a saturation level around 90% binding to the first binding site at 4 μM would require a Kd around 200 nM according to the general law of mass action.

A number of different TTR stabilizers have been identified [16,26,28,30,31,34,44,47,54–63] and several of these have affinity constants below 200 nM and will consequently reach a high level of saturation at the physiological TTR concentration. However, we have recently shown that the efficacy of TTR stabilizers in plasma can be strongly compromised by the presence of other plasma components and that nonspecific binding of drugs might significantly hamper their effect [34].

In present study, we have shown that TBBPA has exceptionally high selectivity for TTR in plasma where a pronounced stabilizing effect on TTR is observed even at concentrations as low as 4 μM. The selectivity of TBBPA for TTR in human plasma is even as good as that of tafamidis, which today is the only approved drug for the treatment of FAP although diflunisal shown similar clinical effects [41].

ITC experiments showed that TBBPA binds to TTR with negative cooperativity having a K_d_1 corresponding to 6 nM and a K_d_2 corresponding to 975 nM.

This is within the same range as tafamidis, which has previously been shown to bind TTR with a K_d_1 of 2 nM and a K_d_2 of 154 nM [11].

Our results raise the question of whether TBBPA can be used as a therapeutic drug. TBBPA is widely used in various consumer goods and building materials and is found in abundance in the environment, both in wildlife [64] and in private households [65], and it has a persistence of days to months in the soil [66,67]. Due to its wide use in different processes and products and the constant human exposure to the molecule, the potential harmful effects of TBBPA have been investigated. These studies have shown that TBBPA has so far not been associated with any major concerns regarding human health risks [39]. TBBPA is thus not regulated worldwide [40]. The European Food Safety Authority (EFSA) recently concluded that TBBPA is not genotoxic and that there are no indications of carcinogenicity and that current dietary exposure is of no concern [68]. The EFSA panel furthermore concluded that the most sensitive endpoint is modifications of thyroid hormones with a benchmark response at 16 mg/kg body weight. A recent investigation demonstrated that prolonged daily oral administration of 100, 300, or 1000 mg·kg^−1^·day^−1^ of TBBPA to Sprague—Dawley rats did not result in increased mortality or the development of neuropathological ailments [68]. Neonatal neural toxicity has previously been reported for brominated flame retardants in the form of diphenyl ethers, but no such toxicity has been observed for TBBPA [69]. A significant reduction of T4 in serum has been observed, but this was not accompanied by a corresponding decrease of the active form of T4, triiodothyronine (T3), and no effects were observed that would indicate perturbed thyroid function such as alterations in T3 and thyroid stimulating hormone levels or overt hypothyroidism [68]. In the same study, the potential harmful effects on reproduction were investigated. Rats were administered oral doses of 0, 10, 100, or 1000 mg·kg^−1^·day^−1^ TBBPA and monitored over two generations. The results showed no significant effects from TBBPA on reproduction, growth, development, morphology, or behavior [68]. There were, however, significant increases in liver weight for those rats receiving 500 and 1000 mg·kg^−1^·day^−1^, but this was not associated with any hepatic histopathological tissue alterations.

The U.S. National Toxicology Program [70] recently reported the results from a 90-day oral toxicity study of TBBPA in B6C3F1 mice administered doses of 0, 10, 50, 100, 500, or 1000 mg·kg^−1^·day^−1^. In that study, some changes in liver, kidney, and spleen weight were observed in addition to an elevated incidence of kidney cytoplasmic vacuolization at the 500 and 1000 mg·kg^−1^·day^−1^ TBBPA dose levels [70,71].

In addition to the acute toxicological effects, the carcinogenic propensity of TBBPA has also been investigated [70,71]. From a two-year analysis performed on Wistar Han rats and B6C3F1/N mice, it was shown that TBBPA at dosages of 500 and 1000 mg/kg given 5 days/week resulted in an approximately 50% increase in the incidence of adenoma and adenocarcinoma of the uterus in the rats [71]. There was also a vague indication that TBBPA treatment at the highest dosage increased the incidence of testicular tumors in rats and hepatic tumors, hemangiosarcomas, and intestinal tumors in male mice [71]. Administration of TBBPA also resulted in an increased incidence of non-neoplastic lesions within the liver and kidney in male mice and within the ovary and uterus in female rats, while both genders had lesions within the forestomach [71]. No carcinogenic effect was seen in female B6C3F1/N mice administered 250 or 500 mg/kg TBBPA [71].

Alterations in estrogen metabolism as well as increased production of free radicals during the metabolism of TBBPA have been suggested as possible modes of action for the increased carcinogenicity [71]. It is also noteworthy that the genotoxicity of TBBPA has consistently produced negative results in Ames tests with strains of *Salmonella typhimurium*, *Escherichia coli*, and yeast cells both with and without metabolic activation [39].

Defining the required degree of tetrameric stabilization to obtain a clinical effect is an important figure to decide a suitable therapeutic dosage. From a report by the European Medical Agency, a plasma concentration corresponding to 3.5–8.5 μM of tafamidis is clinically beneficial and a corresponding steady state level of the drug can be obtained after 14 days of daily administration at subclinical dosage [72]. Considering the IC_50_ for tafamidis in plasma, here found to be around 5 μM, this implies a saturation level corresponding to 31–56% based on the current assay and a plasma concentration of 4μM. Interestingly, these results indicate that although TTR might not be fully saturated *in vivo* tafamidis still exerts a clinical effect. A higher dosage would likely improve the effect even further.

Diflunisal, which has also been identified as a TTR-stabilizing agent with clinical effects *in vivo* [41], has an IC_50_ of 25 μM regarding its ability to stabilize TTR in plasma [34]. The bioavailability of diflunisal is good, however, and the drug is easily taken up when administered orally [73]. A previous study showed that repeated dosing of diflunisal at 250 mg or 500 mg twice per day results in a serum concentration after seven days of 145 μM and 420 μM, respectively, when analyzed 12 hours after the last dose [74]. According to the present assay, this corresponds to a saturation level between 85% and 94%, which is likely the reason for its clinical efficacy. It should be emphasized that although this assay enables a relative comparison between different drugs it involves an inevitable step of denaturation that may shift the observed IC_50_ value and consequently the saturation level in plasma. An exact figure of the saturation levels in plasma should therefore be determined by an independent method.

In analogy to the above examples, a plasma concentration of TBBPA corresponding to 5μM—based on the IC_50_ value—would result in 48% saturation of the high-affinity binding site of TTR. It is therefore interesting to discuss the possibility of obtaining an adequate dosage in humans. TBBPA is absorbed to a considerable extent from the gastrointestinal tract, and similarly to other bisphenols it is metabolized by glucuronyl- and sulfotransferases [75]. Its metabolites are predominantly secreted via the bile and are found in feces. The effective elimination of TBBPA conjugates in bile and enterohepatic circulation however results in a low systemic bioavailability. Serum levels of TBBPA after both single and repeated dosages have so far only been studied in animals, but it can be concluded that oral intake of TBBPA is associated with low bioavailability compared to both tafamidis and diflunisal, which hampers its potential therapeutic use. Discrepancies between different investigations must, however, be noted. In a report by Schauer and co-workers, a single oral dose of TBBPA corresponding to 300 mg/kg body weight in rats resulted in a maximal serum concentration of TBBPA of to 103 μM after 3 h and a half-life around 13 h [75]. The main metabolites were found to be sulfation and, to a lesser extent, glucuronidation of one or both of the two hydroxyl groups of TBBPA [75]. However, a similar study based on a single oral dose corresponding to 250 mg/kg body weight monitored by isotope-labeled ^14^C-TBBPA indicated both a significantly faster decay and a lower uptake with a maximal plasma concentration of only 5.4 μM after 127 min post-dose and a decline consistent with a non-compartmental model having an elimination half-life around 4.5 h [76]. In a third study, oral administration of TBBPA corresponding to 200, 500, and 1000 mg/kg resulted in maximal plasma concentrations of 12, 18, and 31 μM respectively, and half-lives of 7.5–9.5 hours [77]. Interestingly, this study also showed a cumulative effect of TBBPA upon repeated doses, and administration of daily oral doses of TBBPA after 14 days showed an increasing steady-state level in serum [77]. However, although a steady-state level can be reached and a therapeutically relevant plasma concentration can be maintained in the animals through administration of the drug twice per day, this requires a very high dosage that impairs its use as a drug in humans.

Taken together we show in this work that TBBPA is a potent stabilizer of the TTR tetramer and that it displays exceptionally good selectivity in human plasma. TBBPA has been extensively studied in animal models, and it is not linked to adverse cytotoxic effects unless very high doses are given. However, due to rapid modification of its hydroxyl group by the liver enzymes, TBBPA unfortunately has low bioavailability, and this impairs its use as a potential therapeutic drug. The structure of TBBPA nevertheless provides an interesting scaffold in the quest for new and improved stabilizing drugs targeting TTR. We also highlight how the ability to determine the relative selectivity of TTR-stabilizing drugs in plasma provides guidance regarding determination of an adequate dosage in humans.

## Materials and Methods

### Ethical statement

Human plasma, were obtained from the local blood bank (Blodcentralen Umeå; Department of Clinical Immunology and Transfusion Medicine, Umeå University Hospital, SE-901 85 Umeå, Sweden) and only from anonymous donors, precluding the need for informed consent. Institutional review board or ethics committee approval is not required for this study.

### TTR expression and purification

TTR was expressed and purified according to a previously published protocol [46,78]. Briefly, the TTR construct was transformed into *E*. *coli* BL21 cells grown in LB media at 37°C and cultured overnight. The cells were harvested and dissolved in 20 mM Tris buffer pH 7.5, lysed by sonication, and centrifuged at 20,000 × *g* for 30 min. The protein-containing supernatant was loaded onto an anion exchange column (Q-sepharose, GE-healthcare) and eluted with a gradient of NaCl. TTR-containing fractions were further purified by gel filtration (Superdex G-75, 16/60, GE-healthcare) and equilibrated with either PBS for biochemical studies or with minimum essential medium (MEM) for subsequent analysis in the cytotoxicity assay described below.

### Turbidity assay to monitor TTR denaturation at low pH

Tafamidis and TBBPA were dissolved in DMSO. TTR dissolved in PBS at a final concentration of 15 μM was pre-incubated with 15 μM tafamidis, TBBPA, or diflunisal for two hours at room temperature under agitation. In order to prevent microbial growth, all samples and controls were supplemented with 0.05% NaN_3_. Dissociation and subsequent aggregation of TTR was initiated by adding 50 mM sodium acetate at pH 4.5 and incubating at 37°C under agitation conditions. The formation of TTR aggregates was monitored by turbidity measurement at 400 nm in a TECAN Safire plate reader after incubating for 72 h. All experiments were performed five times.

### Cytotoxicity assay

The human neuroblastoma cell line SH-SY5Y was obtained from the European Collection of Cell Cultures (Centre for Applied Microbiology and Research). SH-SY5Y cells were cultured according to a previous report [79] with minor modifications. The cells were grown in MEM and GlutaMax medium (Gibco) and supplemented with 10% (v/v) fetal bovine serum (Gibco), 100 units/mL penicillin, 100 μg/mL streptomycin (Gibco), and 1% non-essential amino acid solution (Gibco). Cultures were maintained in an incubator at 37°C with a humidified atmosphere of 5% CO_2_. Freshly purified TTR (15 μM) in MEM was sterile-filtrated and pre-incubated with TBBPA, tafamidis, or diflunisal (15 μM each) for one hour before being added to the SH-SY5Y cells. The protein-compound solutions were supplemented with 100 units/mL penicillin, 100 μg/mL streptomycin (Gibco), 1% non-essential amino acid solution (Gibco), 2 mM L-glutamine (Gibco), 1% MEM vitamins solution (Gibco), and 1% MEM amino acids solution (Gibco) and incubated with the cells for 48 h at 37°C in a humidified atmosphere of 5% CO_2_. Cytotoxicity was measured using a resazurin reduction test, and cell viability was detected by fluorescence measurement using a TECAN Safire plate reader with excitation at 535 nm and emission at 595 nm. All experiments were performed in triplicate.

### Drug efficacy in human plasma

The plasma assays with the drugs were performed according to a previously published protocol [34]. Briefly, plasma was buffered by the addition of 20 μL phosphate buffer from a 1 M stock solution to reach a final concentration of 20 mM, pH 7.4. The plasma was titrated with tafamidis, TBBPA, or diflunisal from 0 to 32 μM and incubated for 2 h under gentle agitation. To initiate the denaturation of the TTR tetramer, urea was added to a final concentration of 4 M followed by 18 h of incubation at 25°C. In the absence of a stabilizing ligand, this treatment causes dissociation and monomerization of the TTR tetramer. To quantify the formation of monomeric species, the denaturation step was followed by gel electrophoresis using tricine-based SDS-PAGE, which does not dissociate tetramers but prevents monomers from re-associating. The monomeric band on the membrane was visualized by western blot according to standard protocols using a rabbit anti-TTR antibody (anti-prealbumin, DAKO) followed by a horseradish peroxidase-labeled antibody (anti-rabbit HRP, GE Healthcare). Nonspecific binding of antibodies was prevented by the addition of 5% fat-free powdered milk and 0.3% Tween-20. Enhanced chemiluminescence (ECL-prime, GE Healthcare) was used to visualize the bound antibodies. The bands containing the TTR monomer were quantified through densitometric analysis using the ImageJ 1.48 program.

### Isothermal Titration Calorimetry (ITC)

ITC experiments were performed using an Auto-iTC_200_ (MicroCal) at 25°C. TTR concentrations in the cell were 20 μM, and TBBPA was titrated at a 10-fold molar excess. For the control experiment, TBBPA was titrated into the cell with only buffer (PBS with 5% DMSO). For each experiment, 19 automated injections of 2 μL and a duration 0.8 s were performed with 300 s intervals between each injection and with a stirring speed of 1000 rpm. The titrations were done with high feedback and a filter period of 5 s and were repeated twice. Calorimetric data were plotted and fitted using the standard single-site binding model.

### Crystallization of the TTR-TBBPA complex

The protein was crystallized as described previously [80]. Briefly, the purified TTR was dialyzed against 10 mM Na-phosphate buffer with 100 mM KCl (pH 7.6) and concentrated to 5 mg/mL using an Amicon Ultra centrifugal filter device (Millipore, 3 kDa molecular-weight cutoff) and co-crystallized at room temperature with a 5-molar excess of TBBPA using the vapor-diffusion hanging drop method. A drop containing 3 μL protein solution was mixed with 3 μL precipitant and equilibrated against 1 mL reservoir solution containing a range of 1.3–1.6 M sodium citrate and 3.5% *v/v* glycerol at pH 5.5 in 24-well Linbro plates. Crystals grew to dimensions of 0.2 × 0.2 × 0.3 mm^3^ after 5 days. The crystals were cryoprotected with 12% *v/v* glycerol.

### Data collection, integration, and structure determination

The X-ray diffraction data of the TTR-TBBPA complex were collected under cryogenic conditions to 1.40 Å resolution at the European Synchrotron Radiation Facility in Grenoble, France, on beamline ID29 using Pilatus 6M detectors at a wavelength of 0.9137 Å. The diffraction data were processed with XDS [81] and scaled with AIMLESS from the CCP4 software suite [82]. The native structure of TTR (PDB ID: 1F41, [83]) and X-ray data from 30.0–1.40 Å resolution were used in molecular replacement searches with the program PHASER [84]. The TBBPA compound is best defined in the BB' cavity. The model was refined against all of the diffraction data using PHENIX [85]. At the end of structure refinement, anisotropic B-factors were refined resulting in an *R* factor of 14.5% and an *R*_free_ of 18.2% using all data in the range 30.0 Å–1.40 Å. Manual map inspection was performed with COOT [86]. The refined structure comprises residues 10–125 of TTR, 2 TBBPA molecules, 1 sodium ion, and 202 water molecules. There are no Ramachandran outliers. See S1 Table for details of the data collection and refinement statistics. Molecular graphics were produced using CCP4mg [87]. Structure factors and coordinates of the TTR-TBBPA complex have been deposited at the Protein Data Bank (PDB ID: 5HJG).

## Supporting Information

S1 Table Data collection and refinement statistics for the TTRwt-TBBPA complex (PDF)
