# Supplementary material for: Tetrabromobisphenol A Is an Efficient Stabilizer of the Transthyretin Tetramer
Source: PLoS One. 2016 Apr 19;11(4):e0153529. doi: 10.1371/journal.pone.0153529 (PMC4836675; doi:10.1371/journal.pone.0153529)
Supplement: S1 Table — (PDF) [file pone.0153529.s001.pdf]

Supporting Information

Tetrabromobisphenol A is an efficient stabilizer of the transthyretin tetramer

Irina Iakovleva[1], Afshan Begum[2], Kristoffer Brännström[1], Alexandra Wijsekera[1], Jin Zhang[2] Patrik L. Andersson[2], A. Elisabeth Sauer-Eriksson[2], and Anders Olofsson[1]*

[1]Department of Medical Biochemistry and Biophysics, Umeå University, SE-901 87 Umeå, Sweden

[2]Department of Chemistry, Umeå University, SE-901 87 Umeå, Sweden

*Email for correspondence: anders.olofsson@umu.se

Table S1: Data collection and refinement statistics for the TTRwt-TBBPA complex

**Data-collection statistics**	**TTRwt-TBBPA**
Wavelength (Å)	0.9137
Space group	P21212
Unit cell parameters (Å)	a = 42.3, b = 85.3, c = 63.2
Resolution limits (Å)	30.0–1.40 (1.45–1.40)
Total No. of reflections	403324
No. of unique reflections	45696 (4365)
Multiplicity	8.8 (8.7)
Completeness (%)	99.7 (96.7)
R_{sym}	0.064 (0.698)
<I/σ (I)>	17.2 (2.26)
**Refinement and model building statistics**	
Resolution range (Å)	30.00–1.40
R factor (%)	14.5 (19.3)
R free (%)	18.2 (24.9)
No. of protein atoms	2049
No. of water molecules	202
No. of ligand atoms/sodium ions	42/1
**Rms deviations from ideal geometry**	
Bond lengths (Å)	0.009
Bond angles (°)	1.29
**Ramachandran plot**	
Residues in most favored regions (%)	98.0
Residues in allowed regions (%)	2.0
Residues in disallowed regions (%)	0.0
Average B-factor ($Å^2$)	25.3
PDB ID	5HJG

Notes: Data collected from one crystal. Values in parentheses are for the highest-resolution shell. The anomalous difference map was calculated from the above dataset but with unmerged Friedel pairs.
